# Social networks and voter turnout

**DOI:** 10.1098/rsos.230704

**Published:** 2023-10-18

**Authors:** Georgia Kernell, P. J. Lamberson

**Affiliations:** ^1^ Departments of Communication and Political Science, UCLA, Los Angeles, CA, USA; ^2^ Department of Communication, UCLA, Los Angeles, CA, USA

**Keywords:** social network, voter turnout, formal model

## Abstract

This paper develops a model of voter turnout that embeds Riker and Ordeshook’s (1968 *Am. Pol. Sci. Rev.* **62**, 25–42 (doi:10.2307/1953324)) calculus of voting into the context of a social network. In the model, an individual’s expressive benefits to voting depend on the behaviour of their social contacts. We show that there may be multiple equilibria and analyse how these equilibria depend on the structure of the network. We discuss six empirical implications of the model for turnout, some of which suggest novel answers to longstanding puzzles in the turnout literature, such as: why are higher income individuals more likely to vote even in cases when registration costs are low? Why is turnout so difficult to predict? Why does lowering registration costs disproportionately increase turnout among high-income voters? And why do we observe inertia in turnout across elections?

## 1. Introduction

A wealth of empirical evidence supports the hypothesis that voting is contagious. Nickerson [1] finds that when a face-to-face get-out-the-vote message was delivered to one member of a household with two registered voters, 60% of the propensity to vote was passed on to the other registered voter in the household. Using a field experiment conducted on Facebook with 61 million subjects, Bond *et al.* [2] observe that individuals who received a social message encouraging them to vote, featuring the names and pictures of up to six of their Facebook friends who reported voting, were 2.08% more likely to report having voted than those who received an informational message encouraging them to vote with details regarding the location of their polling place. Examining the public voting records for a smaller sample of their subjects revealed that those receiving the social message were 0.39% more likely to vote than users who received the information-only message.

These effects were also partially transmitted to ‘close friends’ of the individuals receiving the treatment.^1^ Sinclair [3] shows that a postcard revealing a registered voter’s voting history was more effective at prompting turnout when the subject’s housemates were habitual voters than when they were not, suggesting a social norm of voting changes the decision calculus.^2^

Despite the abundant evidence that social contacts influence individuals’ decisions to vote, the field lacks a positive political theory explaining how interpersonal influence aggregates to shape broader turnout patterns.^3^ If social influence is a primary driver of turnout, what sorts of regularities would we expect to observe in individual and aggregate behaviour? And, how do those expectations match with existing empirical research? This paper takes a first step in answering these questions by developing a simple model that incorporates social influence into the traditional calculus of voting framework. We suppose that an individual’s decision to vote is influenced by the actions, or expected actions, of their neighbours in a social network. We prove that the model always has at least one stable Bayesian equilibrium (there may be multiple), and the equilibria depend in well-defined ways on the structure of social interaction and the distribution of costs in the population. The model gives rise to a number of predictions relating individual and aggregate turnout to social network structure.

We connect the model’s theoretical predictions with empirical findings by discussing six implications. First, the existence of tipping points and multiple equilibria make predicting turnout difficult, which we conjecture contributes to the high degree of unexplained variance when attempting to predict voting levels from population characteristics [22]. Second, the structure of equilibria can also lead to path dependence over time, resulting in persistent differences in turnout levels across regions or social groups. This finding may partially explain why voting in one election is a strong determinant of subsequent behaviour at both the aggregate and individual level [23]. Third, individuals with more social contacts participate in greater numbers. As we discuss below, this implication may help to explain why more educated and higher income individuals vote at higher rates, even in the absence of substantial registration requirements [24,25]. Fourth, lowering the costs of voting increases turnout. Besides this straightforward prediction, we also show how social influence may explain a less intuitive empirical relationship: lowering costs disproportionately increases turnout among high socio-economic status voters [26,27]. Fifth, more connected networks lead to higher turnout. This implication is consistent with the observation that participation is greater in high-salience elections, which probably give rise to an increase in political discussion and consequently more connected political discussion networks [28]. Furthermore, the relationship between network connectivity and turnout may contribute to differences in turnout across communities, time and social groups that are unexplained by more commonly observed demographic characteristics. Sixth, we discuss how increasing individual heterogeneity with respect to the number of contacts affects turnout.

## 2. Modelling social influence in the turnout decision

Building on Downs [29], Riker & Ordeshook [30] propose a calculus of voting model in which an individual receives rewards *R* from voting given by

R=PB+D−C,

where *P* is the probability that their vote will make or break a tie, *B* is the difference in benefits if their preferred candidate wins, *D* represents benefits from the act of voting alone regardless of the election outcome and *C* represents the costs of voting. A person votes if *R* > 0. Since the probability that a single vote is pivotal is essentially zero in any large election, we drop the *PB* term from the analysis, leaving

2.1 R=D−C.

This paper examines the effects of one possible component of *D*: the expected actions of other individuals. Suppose that an individual *i* has *d*_*i*_ social contacts and expects a fraction of them, *x*, to vote. In the language of network science, *d*_*i*_ is agent *i*’s *degree*. Let *U*(*d*_*i*_, *x*) denote *i*’s expected socially derived benefits from voting. Define *E*_*i*_ as any remaining expressive benefits, so *D*_*i*_ = *U*(*d*_*i*_, *x*) + *E*_*i*_.

Substituting into equation (2.1), the rewards to voting for individual *i* become *R*_*i*_ = *U*(*d*_*i*_, *x*) + *E*_*i*_ − *C*_*i*_, and agent *i* will choose to vote if

$$U(d_{i},x)\geq C_{i}-E_{i}.$$

We refer to *C*_*i*_ − *E*_*i*_ as *i*’s *net costs*.

The model is flexible in that there is no fixed functional relationship between the actions of a person’s social contacts and the resulting benefits. The only restriction we impose is that the function *U* be non-decreasing in *x* and *d*, implying that an individual receives more utility from voting when a greater fraction of their social contacts vote or when they expect the same fraction of their contacts to vote but there are more of them, and therefore they expect to have a greater absolute number of voting contacts. These assumptions are consistent with the argument that social pressure comes from a desire to conform with the actions of our contacts, and the empirical evidence discussed in the introduction supports this.

Beyond these restrictions, the model allows for any possible relationship that depends on an agent’s degree and the fraction of those contacts that they expect to vote. For example, the contribution of social influence to the rewards from voting might be a simple linear function of the number of voting contacts: *U*(*d*, *x*) = *α* + *β* · *dx*. Or, an individual might prefer to conform with the majority of their contacts:

$$U(d,x)=\left\{ \begin{array}{cc} \alpha \; & x\geq 0.5\;\\ 0\; & x<0.5\; \end{array}\right..$$

### 2.1. Information

Even in small networks the model described in the previous section admits too many equilibria to systematically analyse. Following a number of other formal models of strategic behaviour in networks, we introduce a partial information structure to help solve the equilibrium selection problem [31–41]. Specifically, we assume that individuals know the number of other agents with whom they expect to interact but do not know their specific identities. Given this information, agents expect their contacts to act like random draws from the set of *connections* in the population.

It is important to note that drawing from the set of possible connections is not the same as drawing from the set of agents, because in the former case an individual is more likely to contact a high-degree agent. Specifically, the probability a randomly chosen contact has degree *d* is given by the *neighbours’ degree distribution*, $\tilde{P}(d)\;:=dP(d)/\bar{d}$, where $\bar{d}$ is the average degree over all agents [42]. The resulting probability that a randomly chosen contact votes is

$$x=\sum_{d}\tilde{P}(d)\pi_{d},$$

where *π*_*d*_ is the fraction of degree *d* agents who vote. We call *x* the *link-weighted fraction of voters*. Note that this is the same use of the variable *x* as before—the link-weighted fraction of voters is the fraction of contacts that any given individual expects to vote.

An agent with *d* contacts votes as a best response if their net costs are less than *U*(*d*, *x*), so the fraction of degree *d* agents that vote as a best response when the current link-weighted fraction of voting agents in the population is *x* is given by

2.2 G(d,x) :=F(U(d,x)),

where *F* is the cumulative distribution of net costs.

Then at an equilibrium

2.3 $$x=\sum_{d}\tilde{P}(d)G(d,x).$$

Equation (2.3) characterizes symmetric Bayesian Nash equilibria of the game.^4^ For the purpose of analysing these equilibria, the game is completely specified by the triple, {*P*, *F*, *U*}, of the degree distribution, net cost distribution and socially derived utility function. We call any such triple *a social voting game.* Abrams *et al*. [21] also analyse the Bayesian Nash equilibria of a similar game-theoretic model of voter turnout; however, their group-based model focuses on the predictors of social approval for voting and the possibility of strategic social sanctioning of non-voters, while we concentrate on the role of network topology.

Below, we analyse the symmetric Bayesian equilibria of the game, as determined by equation (2.3), and the dependence of the equilibria on the distribution of costs and agents’ degrees. Such an equilibrium always exists, and for any fixed distribution of costs and degree there may be multiple equilibria.

### 2.2. Dynamics

One interpretation of the equilibria defined in the previous section is that they result from all of the agents simultaneously reasoning about one another’s best response strategies. A different, perhaps more believable, interpretation is that these equilibria are potential endpoints of a dynamic process in which agents meet and share their voting intentions with their social contacts and then update based on those conversations. For the remainder of the paper, we will adopt this second interpretation.

To be precise, suppose that at some time zero, well in advance of a future election, a fraction *x*_0_ of the population plans to vote. Each agent, *i*, contacts *d*_*i*_ other agents and learns of their voting intentions. As a result, *i*’s expected utility from voting is now

2.4 $$R_{i,0}=U(d_{i},x_{0})+E_{i}-C_{i}.$$

Any agent for whom equation (2.4) is positive will subsequently plan to vote; conversely, any agent for whom equation (2.4) is negative will not. Following the same logic described in the previous section, the resulting expected turnout fraction at time one as a best response to the observations from time zero is $x_{1}=\sum_{d}\tilde{P}(d)G(d,x_{0}).$ Similarly, at time *t*, $x_{t}=\sum_{d}\tilde{P}(d)G(d,x_{t-1}).$ The Bayes–Nash equilibria described in the previous section are the long-run equilibria of this dynamic discussion process.

We think of this dynamic process as capturing the political discussion in the months and weeks leading up to an election. As people discuss their political views with one another, they update their beliefs about the actions they expect their social contacts to take, and consequently revise their own intentions. Over time, the fraction of voters converges to an equilibrium as defined in §2 above. The particular equilibrium that is ultimately reached may depend on the initial fraction of voters.

## 3. Results

We begin by discussing the existence and structure of equilibria in the model. Define *ρ*(*x*) by

3.1 $$\rho (x)=\sum_{d}\tilde{P}(d)G(d,x).$$

Then fixed points of *ρ* correspond to Bayesian Nash equilibria. We call *ρ* the *dynamic function* associated with the underlying model parameters and note that in the dynamic interpretation described in the previous section, *x*_*t*_ = *ρ*(*x*_*t*−1_).

**Theorem 3.1.**

*Every social voting game*, {*P*, *F*, *U*}, *has at least one symmetric Bayesian Nash equilibrium*.

**Proof.**

Under the assumption that *U* is increasing in *x* for any fixed *d*, *ρ* : [0, 1] → [0, 1] is also monotonically increasing. Thus, by Tarski’s theorem, *ρ* has at least one fixed point [43]. ▪

Mathematically, these equilibria can be categorized as stable or unstable. Stable equilibria are robust to small perturbations, while unstable equilibria are not. Given that political discussions and turnout can be affected by random events in the real world, stable equilibria will be more interesting in the sense that they represent more likely outcomes. But, unstable equilibria are also worthy of attention because they can act as ‘tipping points’ that separate different stable outcomes [44].

**Figure 1.**
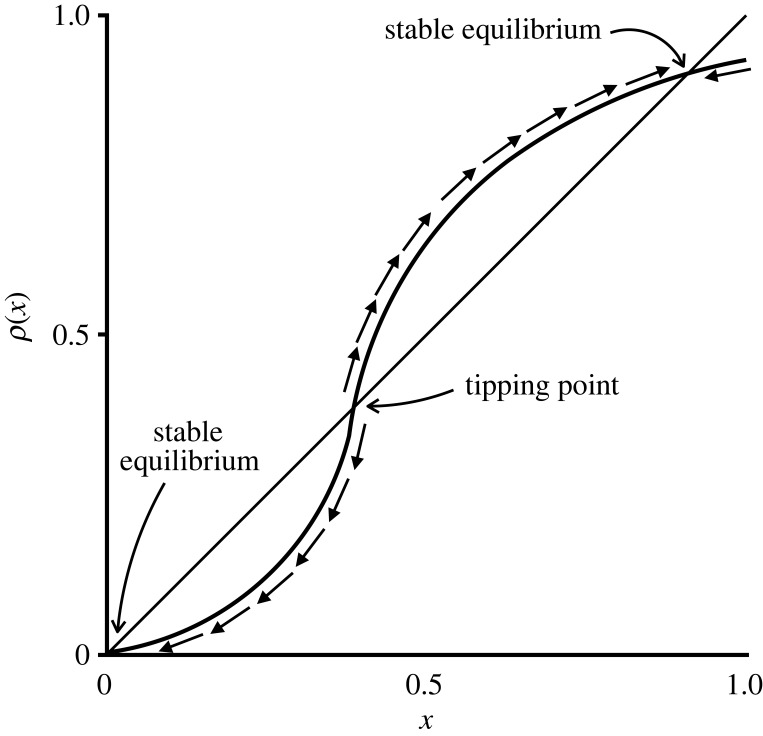
An example of the function *ρ* illustrating the corresponding best-response dynamics and equilibria.

Figure 1 illustrates an example of the function *ρ*. When *ρ*(*x*) > *x*, meaning that the curve is above the $45^{\circ }$ line, turnout is higher at *t* than at *t* − 1. Conversely, when *ρ*(*x*) < *x*, and the curve is below the $45^{\circ }$ line, fewer individuals plan to vote at *t* than at *t* − 1. A point where the curve goes from under to over the $45^{\circ }$ line (moving from left to right) is an unstable equilibrium, also known as a tipping point—while the exact point itself is an equilibrium, any slight deviation will send the population away from it. A point where the curve goes from over to under the $45^{\circ }$ line is a stable equilibrium—all nearby points converge to it. It is possible for *ρ* to just touch the $45^{\circ }$ line without crossing it, resulting in a degenerate fixed point. However, such a degenerate fixed point can be removed by an arbitrarily small perturbation of the model parameters, and so we assume that no such degenerate fixed points exist.

The following theorem characterizes the structure of the equilibria.

**Theorem 3.2.**

*Every social voting game*, {*P*, *F*, *U*}, *has at least one stable equilibrium. The set of fixed points alternates between stable and unstable, where both the lowest and highest equilibria are always stable*.

**Proof.**

Let *ρ* be the dynamic function associated with the social voting game {*P*, *F*, *U*}. Since *ρ*(0) ≥ 0, either zero is a stable equilibrium, or the first non-zero equilibrium is stable. Similarly, since *ρ*(1) ≤ 1, either one is a stable equilibrium, or the greatest equilibrium less than one is stable. The fact that equilibria alternate between stable and unstable follows from the definition, continuity of *ρ*, and the assumption of no degenerate equilibria. ▪

The next theorem is a straightforward consequence of the assumption that *U*(*d*, *x*) is increasing in *d* for all *x*.

**Theorem 3.3.**

*At any equilibrium of any social voting game, agents with higher degree have a higher probability of voting*.

**Figure 2.**
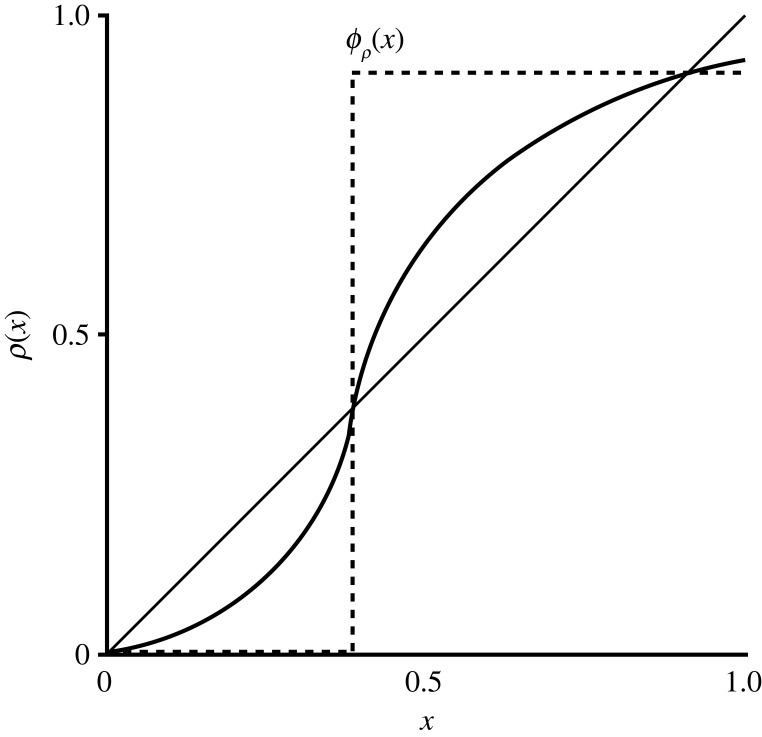
An example of the equilibrium function $\phi_{\rho }$ associated with *ρ*.

Now we turn to understanding which conditions generate greater turnout at equilibrium. This is less straightforward than it seems because for a given set of parameters there may be multiple equilibrium levels of turnout. To better describe this dependence, we define a function $\phi_{\rho }\;:\;[0,1]\to (0,1)$, which we call the equilibrium function of *ρ*. For any *x* ∈ [0, 1] with *ρ*(*x*) < *x*, define $\phi_{\rho }(x)$ to be the largest stable equilibrium of *ρ* that is less than *x*. For any *x* ∈ [0, 1] with *ρ*(*x*) ≥ *x*, define $\phi_{\rho }(x)$ to be the smallest stable equilibrium of *ρ* that is greater than or equal to *x*. Intuitively, the equilibrium function returns the likely endpoint when the discussion process begins at *x*. See figure 2 for an example. Then we make the following definition:

**Definition 3.4.**

A dynamic function *ρ* produces greater turnout than a dynamic function $\tilde{\rho }$ if $\phi_{\rho }(x)\geq \phi_{\tilde{\rho }}(x)$ for all *x* ∈ [0, 1].

Intuitively, this means that for any given initial point, the long-run equilibrium under *ρ* will be greater than that under $\tilde{\rho }$. With this definition in hand, we can compare the effects of different sets of parameters. We begin with the following useful lemma. The lemma is analogous to Lemma 1 in [38].

**Lemma 3.5.**

*If* $\rho (x)\geq \tilde{\rho }(x)$ *for all* *x*, *then* *ρ* *produces greater turnout than* $\tilde{\rho }$.

We refer the interested reader to [38] for details of the proof. The intuition is that as *ρ* increases, stable equilibria increase, unstable equilibria decrease, and when a stable and unstable equilibrium meet, they both vanish.

**Theorem 3.6.**

*If the net cost distribution* *F first-order stochastically dominates the cost distribution* *F*′, *then* *F*′ *produces greater turnout then* *F*.

**Proof.**

Substituting (2.2) into (3.1) gives

$$\rho (x)=\sum_{d}\tilde{P}(d)F(U(d,x)).$$

Let $\tilde{\rho }(x)$ be the corresponding dynamic function with *F* replaced by *F*′. By the definition of first-order stochastic dominance, *F*(*U*(*d*, *x*)) ≤ *F*′(*U*(*d*, *x*)), so $\rho (x)\leq \tilde{\rho }(x)$. Thus, by lemma 3.5, *F*′ produces greater turnout then *F*. ▪

**Theorem 3.7.**

*If the neighbours’ degree distribution* $\tilde{P}$ *first-order stochastically dominates the neighbours’ degree distribution* ${\tilde{P}}^{\prime }$, *then* $\tilde{P}$ *produces greater turnout then* ${\tilde{P}}^{\prime }$.

**Proof.**

From equation (3.1),

$$\rho (x)=\sum_{d}\tilde{P}(d)G(d,x).$$

From equation (2.2), *G*(*d*, *x*) = *F*(*U*(*d*, *x*)), which is increasing in *d*, so by the definition of first-order stochastic dominance, $\sum_{d}\tilde{P}(d)G(d,x)\geq \sum_{d}{\tilde{P}}^{\prime }(d)G(d,x)$. Thus, by lemma 3.5, $\tilde{P}$ produces greater diffusion than ${\tilde{P}}^{\prime }$. ▪

**Theorem 3.8.**

*Suppose that the degree distribution* *P* *is a mean preserving spread of the degree distribution* *P*′. *If* *G*(*d*, *x*) *is linear or convex as a function of* *d*, *P* *will generate greater turnout than* *P*′.

**Proof.**

If *P* is a mean preserving spread of the degree distribution *P*′ then *P*′ second-order stochastically dominates *P*. Second-order stochastic dominance implies

$$\sum_{d}u(d)P^{\prime }(d)\leq \sum_{d}u(d)P(d),$$

for every non-decreasing convex function u : R→R. If *G*(*d*, *x*) is linear or convex as a function of *d*, then $dG(d,x)/\bar{d}$ is convex as a function of *d*, so

$$\sum_{d}{\tilde{P}}^{\prime }(d)G(d,x)=\sum_{d}P^{\prime }(d)dG(d,x)/\bar{d}\leq \sum_{d}P(d)dG(d,x)/\bar{d}=\sum_{d}\tilde{P}(d)G(d,x).$$

Thus, by lemma 3.5, *P* produces greater turnout than *P*′. ▪

## 4. Implications

Having established a number of theoretical predictions of our model, we now turn to the empirical implications of those results.

### 4.1. Tipping points and unpredictability

The first implication follows from the structure of stable and unstable equilibria in the model described in theorems 3.1 and 3.2. The existence of multiple equilibria and tipping points can lead to highly unpredictable behaviour.

**Implication 4.1.**

Small differences in the initial conditions or the influence of exogenous factors may lead to large changes in turnout.

In a recent meta-analysis of 44 articles on turnout, Frank and Martínez i Coma [45] found over 120 potential predictors of voter turnout in the literature. After running 15 million regressions using data from 70 of these predictors, they found 22 that were robustly associated with turnout. And yet, despite the incredible effort put towards explaining turnout, a great deal of variance remains [22,46]. Implication 4.1 may help explain why. Tipping points and multiple equilibria result in a nonlinear relationship between the factors that influence the decision to vote at the individual level and collective levels of turnout.

**Figure 3.**
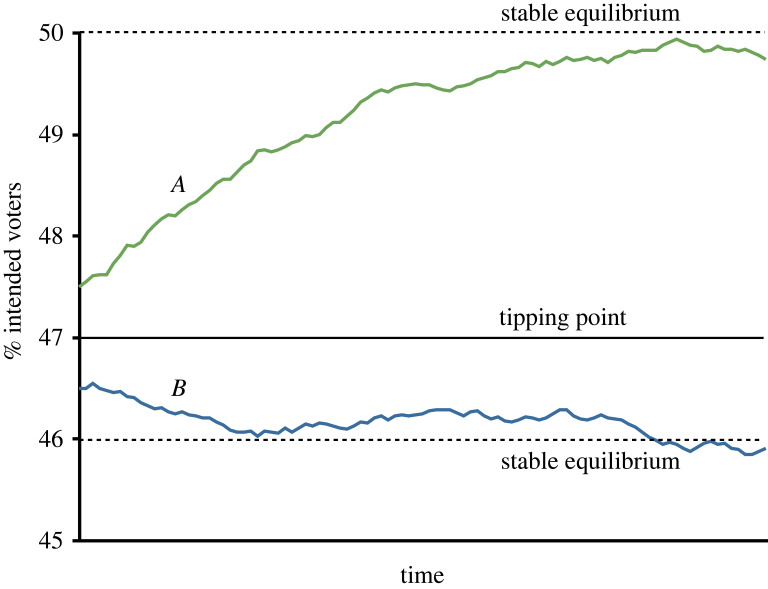
A hypothetical evolution of turnout percent in two communities, *A* and *B*, in the run-up to an election. Community *A* begins with turnout just above a tipping threshold and community *B* begins with turnout just below the tipping point. By the time of the election, the two initially similar communities have converged to very different equilibria.

Figure 3 illustrates an example. Suppose that in the absence of social influence, we could predict turnout exactly using variables such as age, education, party identification, election closeness and so on. Consider two communities, *A* and *B*, with nearly identical predicted turnout. If turnout in community *A* is slightly above a tipping point there may be enough social pressure to ‘get the ball rolling’ and increase turnout even more, providing more social pressure, and increasing turnout and so on in a reinforcing feedback until the next higher stable equilibrium is reached. If, on the other hand, community *B* has not crossed the next tipping threshold, either because initial turnout is slightly lower in community *B* or because differences in the two communities' social networks result in different tipping points, social pressure will not push turnout in *B* higher. As a result, the two communities, which initially appeared similar, may wind up at very different equilibria.

The existence of tipping points may also affect turnout over time within a community in the run-up to an election. If, say, a mobilization effort temporarily pushes the number of expected voters past a threshold, then the effect of communication can take over, pushing the population to an even higher level of turnout. Conversely, a downward shock, such as a political scandal, might drop turnout to a point where insufficient social pressure leads to still further turnout declines.

### 4.2. Basins of attraction and stability

While tipping points can magnify small changes in turnout, in between those tipping points lie basins of attraction where any perturbations are counteracted by the magnetism of stable equilibria. This leads to our second implication:

**Implication 4.2.**

Community levels of turnout across elections, and expected turnout in the run-up to an election, will tend to be stable over time.

At first, this implication appears to run counter to Implication 4.1. How can turnout be both stable and unpredictable? The key difference is in what predictors we use in our forecast. Implication 4.1 applies to turnout forecasts based on individual characteristics—communities with similar populations may end up at very different levels of turnout, while others with very different populations may wind up with nearly identical turnout. On the other hand, Implication 4.2 applies to forecasting turnout with turnout from other elections or time periods in the same population. To make an analogy, it might be quite difficult to predict the weather using measurements like humidity, temperature and air pressure, but it is likely to be similar to yesterday.

As discussed in the previous section, tipping points can magnify the effect of a mobilization effort if the intervention pushes voting across a tipping threshold. Conversely, any mobilization effort that fails to push turnout across a tipping point may only exhibit a temporary bump as there is insufficient social pressure to maintain the change. This tendency to be drawn back towards a stable equilibrium may help explain the significant difficulty in inducing persistent mobilization [47–49].

Similarly, basins of attraction also provide regularity within communities across elections. If potential voters’ initial turnout expectations are based on levels of turnout from previous elections, communities with low turnout in the past will tend to continue to have low turnout in the future. Because they start out with low turnout expectations, it will be difficult for them to cross a higher tipping point to converge to a higher level of turnout. Similarly, communities with high turnout in the past will continue to have high turnout. For example states like Hawaii and West Virginia are consistently among the lowest turnout states in presidential elections while states like Minnesota, Wisconsin and Oregon have consistently high turnout levels. While path dependence can maintain persistent turnout differences between communities over time, it may also lead an exogenous turnout change in one election to carry over to future elections. Green & Shachar [23] observe that turnout in a given election appears to be a strong determinant of turnout in a subsequent election. They argue that this turnout inertia results from a sort of psychological comfort with the voting process formed at the individual level (see also [50]). Our model of social influence provides an alternative explanation for the observation of turnout inertia.

### 4.3. Number of contacts

The implication of theorem 3.3 is clear:

**Implication 4.3.**

Individuals with more social contacts are more likely to vote.

In the model, people with more contacts are more likely to vote because they feel more social pressure to do so. This implication is supported by a range of empirical studies that find correlations between individual political conversation and participation. For example, a laboratory experiment by Schram & Sonnemans [8] demonstrates that increasing opportunities for communication lead to greater political participation. La Due Lake & Huckfeldt [51] find that both the size of an individual’s network and the frequency of political interactions within that network are associated with a person’s likelihood of voting, as well as a number of other presumably more costly activities such as attending meetings and rallies or donating money to a party or campaign.^5^

This implication may also contribute to turnout disparities by education and income [24,53,54]. McPherson *et al*. [55] find that education has a significant positive association with network size,^6^ and Weatherford [57] finds that socio-economic status is associated with an increase in the fraction of an individual’s contacts with whom they discuss politics. If higher education and income individuals have a larger number of contacts, our model predicts they will be more likely to vote. This effect operates independently of any cost differences, so even if informational or registration requirements are equal, variation in network size could partially explain observed differences in turnout across income and education levels. As we discuss in Implication 4.5, this effect may be reinforced at the community level.

Evidence from a natural experiment provided by the introduction of optional voting by mail in Switzerland [12] finds that while optional voting by mail increased turnout overall, the effect differed significantly by community size. By minimizing the social pressures associated with going to the polls in person, smaller communities actually saw a decrease in turnout. Funk [12] posits that ‘in small communities, people know each other better and gossip about who fulfils civic duties and who doesn’t', and as a result, ‘social benefits were particularly high in this type of community'. Voting by mail eliminates those social benefits. Supporting this argument, Carreras & Bowler [58] (discussed in §4.5 below) find that individuals in rural Latin American communities tend to have higher levels of social capital.

### 4.4. The cost distribution

The implication of theorem 3.6 is also straightforward:

**Implication 4.4.**

Lower costs of voting lead to higher turnout.

Costs in the model are distributed randomly across potential voters according to the distribution *F*. Suppose that, for example due to a change in registration laws, the costs of voting are reduced. Mathematically, we can capture this by changing to a new cost distribution, *F*′, where *F*(*c*) ≤ *F*′(*c*) for all *c*. The distribution *F* is said to *first-order stochastically dominate* the distribution *F*′. Theorem 3.6 shows that lowering the cost of voting, in the sense of first-order stochastic dominance of the distribution of costs, increases turnout. This prediction is unsurprising, but it is reassuring that it continues to hold when social influence is incorporated into the model.

The effect of lowering costs on turnout is not spread equally across the population. Moving from a cost distribution *F* to a lower distribution *F*′ disproportionately increases the probability of voting among high degree voters. Combining this observation with the correlations between degree and socio-economic status discussed in §4.3, the model predicts that lowering voting costs will increase turnout more among high education and high economic status individuals. This counterintuitive implication is exactly the ‘perverse consequence of electoral reforms’ described by Berinsky [27, p. 1]. For example, Karp & Banducci [26] and Berinsky *et al.* [59] find that switching to postal voting in Oregon led to a disproportionate increase in voting among higher socio-economic status individuals.

### 4.5. Network connectivity

Theorem 3.7 applies the notion of first-order stochastic dominance to the degree distribution, implying that:

**Implication 4.5.**

More connected networks lead to higher levels of turnout.

This implication applies to differences in networks across time, geography or social community, and may explain or reinforce other observations about turnout. For example, electoral competitiveness is generally correlated with turnout [60,61]. Conventional wisdom expects politicians to exert greater effort to mobilize voters in close, competitive elections [60–62]. Cox *et al.* [63] argue that this relationship is conditional on a politician’s social capital; those with a stronger social network are more effective at, and thus more likely to put effort into, mobilizing voters. Building on this argument, we suggest that in close elections the entire political discussion network should become more connected. Whether through increased campaign spending or greater news coverage, close elections give rise to more pre-election chatter, and thus may boost turnout.

As in Implication 4.3, differences in the connectedness of political discussion networks may also contribute to variation in turnout across social groups, but in this case at the group rather than individual level. People form social connections with individuals that are similar to them [64]. Thus, social influence coupled with observed differences in network density across different groups may help to explain turnout differences across those groups. Harell [65] makes this argument in explaining why women turn out as much or more than men in many advanced industrial democracies despite having lower average education or income. Using survey data from Canada, the author finds that women have greater levels of ‘informal’ social capital (i.e. unpaid volunteer work provided outside of a formal organization), and these social connections increase turnout. Similarly, Carreras & Bowler [58] find that residents in rural Latin American communities have higher levels of social capital because they participate in professional and religious meetings at greater rates, come from larger and more cohesive families, and enjoy closer ties with their neighbours. The authors show that rural residents turn out at higher rates than those from urban communities, and the difference in social capital explains the turnout disparity.

Using data from the General Social Survey, Rolfe [14, p. 134] argues that ‘there appear to be two distinct subgroups in the American population: low-education respondents whose close friends are drawn exclusively from a community of low-education members, and respondents of diverse educational backgrounds whose personal networks include one or more individuals with a college education'. Rolfe finds that individuals in the latter group have larger personal networks and turn out at a higher rate. Moreover, the author shows that once we control for social context ‘individual education appears to have little, if any, independent influence on an individual’s turnout probability’ [14, p. 146], but ‘both social world and personal network properties [degree] contribute independently to turnout probability’ [14, p. 147]. In other words, an individual’s number of contacts, as in Implication 4.3, and the overall connectedness of their community, as in Implication 4.5, are associated with higher turnout.

### 4.6. Degree heterogeneity

Theorem 3.8 considers the effect of heterogeneity in the number of contacts across agents. The corresponding implication is:

**Implication 4.6.**

If the marginal increase in the probability of voting caused by adding one additional contact is constant or increases with the number of contacts, then an increase in degree heterogeneity across individuals increases turnout.

To gain some intuition for this implication, consider the following example. Suppose that the distribution of costs is uniform (with support larger than [0, max *U*(*d*, *x*)]) and that the benefits function *U*(*d*, *x*) is convex as a function of *d*. Then increasing the heterogeneity in the number of contacts across individuals will increase turnout.

Many commonly studied network types can be ordered in terms of mean preserving spreads. In a *regular* network, every individual has the same number of connections. A random network in which any two nodes are linked with probability *p* will have a degree distribution that follows a Poisson distribution (in the limit as the number of nodes increases). Such a network is called an *Erdös–Rényi network* or a *Poisson random network*. In an Erdös–Rényi network, the modal number of connections equals the mean number of connections, and there is a decreasing probability of having higher or lower number of connections (similar to a normal distribution). By contrast, consider a network that is sequentially built up by connecting new nodes to the existing network with a single edge one at a time. Suppose that with a fixed probability *m* the new node connects to a given existing node with probability proportional to the degree of the existing node, and with probability 1 − *m* the new node connects to one of the existing nodes selected uniformly at random. Such a network will have a power law degree distribution and is called a *scale-free* or *power law* network. In contrast to an Erdös–Rényi network, in a power law network most nodes have very few connections, and a few nodes have a large number of connections—like the hub and spoke system of an airline. Many real social networks look roughly like this; some individuals have many contacts, but most have relatively few connections. For a fixed average degree, the degree distribution of a power law network is a mean preserving spread of an Erdös–Rényi network, which is a mean preserving spread of a regular network [42]. Thus, if the conditions of Implication 4.6 are satisfied, then we would expect greater turnout in a community with a power law network than in a community with an Erdös–Rényi network and greater turnout with an Erdös–Rényi network than with a regular network.

Like Implication 4.5, this result may shed some light on changing patterns of turnout over time and across populations, but there is less data available on the heterogeneity of connections from which to draw inferences. A more immediate application for this result is to future models of voting and political behaviour that involve social networks, which are often based on an assumed underlying social network of one of the types discussed above. As Implication 4.6 shows, the type of network employed can affect the resulting outcome even if the mean degree is fixed.

## 5. Conclusion

This paper demonstrates the potential importance of social influence in the calculus of voting and discusses six resulting implications for voter turnout. The model predicts the existence of multiple stable equilibria separated by tipping points, the highest degree individuals will be most likely to vote, reducing the costs of voting will increase turnout but will do so disproportionately across individuals according to their level of political engagement, turnout will be highest in the most connected networks, and heterogeneity in the number of connections across individuals should shape turnout in predictable ways. These results can help us to understand patterns in turnout across communities, social groups and over time. As Abrams *et al.* find, ‘social influences through groups can make nearly all the difference in whether a person goes to vote or stays at home’ [21, p. 255].

The theoretical predictions of social network models are especially important given the difficulty of gathering data on the relationship between network structure and behaviour. While studies such as those conducted by Nickerson [1], Bond *et al.* [2] and Sinclair [3] provide evidence that social pressure affects turnout at the individual level, the premise of this line of research is that individual turnout decisions interact in non-trivial ways [5]. Measuring how social structure affects turnout at the population level requires data from multiple elections with controlled differences in the social network. Such accurate social network data are inherently difficult to obtain, especially across many networks. The theory presented here helps identify which data are most important to collect and may be of particular use in explaining variation in turnout across contexts and over time.

In our model, we neglect the instrumental term, *PB*, from the classic voting calculus because the chance any given individual is pivotal is vanishingly small in most elections. An alternative explanation for voter turnout focuses on group membership and relies on the assumption that the probability of the group being pivotal is sufficiently large [61,66–72]. Social influence might also lead an individual’s action to have a non-trivial chance of swinging the election if their choice to vote creates a cascade of voting among their peers. Interestingly, the resulting effect on *P* may be inversely related to that on *D*; if everyone around someone is voting, they have the least potential for social influence. We leave exploration of this possibility for future research.

While our model examines social network effects on turnout, social influence probably shapes a wide array of political behaviours. In addition to turnout, Sinclair [3] provides evidence for social network effects on campaign giving, vote choice and party identification. Others have discovered network effects in regulatory enforcement [73], the spread of democracy [74] and the adoption of policy innovations [75]. Each of these arenas could benefit from modelling efforts like the one here to illuminate the role that network structure plays in determining individual and collective outcomes. However, as past research has established, details of the influence process matter; simple contagion models [32], social learning models [38], threshold models [76] and game-theoretic models [37] all predict different relationships between network structure and collective behaviour. Thus, it is critical for any future models of network effects in politics to take seriously the nuances of social influence in each domain.

## Data Availability

This article has no additional data.

## Footnotes

1 Close friends were defined as those in the eightieth percentile or higher of frequency of interactions.

2 For a sampling of additional literature supporting the social contagion of voting, see [4–15].

3 While there are many models of social contagion in networks more broadly (see [16] for a review of recent network diffusion models in economics), few address voter turnout specifically. Granovetter’s foundational threshold model [17] mentions turnout as one possible application. More recent notable exceptions are the model by Amaro de Matos & Barros [18] and agent-based models such as those developed by Fowler & Smirnov [19], Fowler [20] and Rolfe [14]. The model of Abrams *et al*. [21], discussed in more detail below, bears the most similarity to ours.

4 By symmetric, we mean that any two agents with the same degree and costs will play the same strategy.

5 At the community level, Atkinson & Fowler [52] report a contradictory finding: community activities that generate social capital appear to decrease turnout. The authors suggest three possible explanations for their outlying finding. Increased social interactions might expose citizens to conflicting views, creating uncertainty that leads to less turnout. Alternatively, the events studied (saint’s day fiestas) may take time away from participants that otherwise could have been used for political activities. Or, the fiestas may have provided citizens with the sense of civic engagement and fulfilment that they might otherwise have sought from voting. The latter two explanations do not contradict our theory, but the first does.

6 In McPherson *et al.*’s data, one individual is linked to another if the first person ‘discusses important topics’ with the second person. Klofstad [56] shows that network sizes inferred from questions probing discussion of important matters are similar to those that ask about political discussions specifically.
